# Association between medial gastrocnemius muscle-tendon unit architecture and ankle dorsiflexion range of motion with and without consideration of slack angle

**DOI:** 10.1371/journal.pone.0248125

**Published:** 2021-03-05

**Authors:** Kosuke Hirata, Hiroaki Kanehisa, Naokazu Miyamoto

**Affiliations:** 1 Research Fellow of Japan Society for the Promotion of Science, Tokyo, Japan; 2 Graduate School of Engineering and Science, Shibaura Institute of Technology, Saitama, Japan; 3 Department of Sports and Life Sciences, National Institute of Fitness and Sports in Kanoya, Kagoshima, Japan; 4 Faculty of Sport and Health Science, Ritsumeikan University, Shiga, Japan; 5 Faculty of Health and Sports Science, Juntendo University, Chiba, Japan; Tokai University, JAPAN

## Abstract

Joint flexibility is theoretically considered to associate with muscle-tendon unit (MTU) architecture. However, this potential association has not been experimentally demonstrated in humans in vivo. We aimed to identify whether and how MTU architectural parameters are associated with joint range of motion (RoM), with a special emphasis on slack angle. The fascicle length, pennation angle, tendinous tissue length, MTU length, and shear modulus of the medial gastrocnemius (MG) were assessed during passive ankle dorsiflexion using ultrasound shear wave elastography in 17 healthy males. During passive dorsiflexion task, the ankle joint was rotated from 40° plantar flexion to the maximal dorsiflexion joint angle at which each subject started experiencing pain. From the ankle joint angle-shear modulus relationship, the angle at which shear modulus began to rise (slack angle) was calculated. Two dorsiflexion RoMs were determined as follows; 1) range from the anatomical position to maximal angle (RoM_anat-max_) and 2) range from the MG slack angle to maximal angle (RoM_slack-max_). The MTU architectural parameters were analyzed at the anatomical position and MG slack angle. The resolved fascicle length (fascicle length × cosine of pennation angle) and ratios of resolved fascicle or tendinous tissue length to MTU length measured at the MG slack angle significantly correlated with the RoM_slack-max_ (r = 0.491, 0.506, and -0.506, respectively). Any MTU architectural parameters assessed at the anatomical position did not correlate with RoM_anat-max_ or RoM_slack-max_. These results indicate that MTUs with long fascicle and short tendinous tissue are advantageous for joint flexibility. However, this association cannot be found unless MTU architecture and joint RoM are assessed with consideration of muscle slack.

## Introduction

Joint flexibility is an essential physical characteristic related to athletic performance [1, 2] and injury risks [3, 4]. Thus, the identification of factors influencing joint flexibility can help advance athletic performance and reduce injury risks. Joint flexibility is generally defined as the ability to rotate a joint widely without suffering pain or discomfort [5]. Although joint flexibility is multifactorial, stretch-induced changes in muscle-tendon unit (MTU) architecture (i.e., fascicle length, pennation angle, and tendinous tissue length) are considered as the primary contributors to joint flexibility [6, 7]. Because MTU extensibility, which denotes the maximal stretch-induced change in MTU length [8], is affected by the MTU architecture [9, 10], the MTU architecture per se may be associated with joint flexibility. For example, it is theoretically [11, 12] and experimentally [6, 13] implied that long fascicle is advantageous to joint flexibility. Additionally, considering the facts that muscle is much more compliant than tendinous tissue under resting condition [14] and that MTU length is limited in vivo, it is rational to assume that muscle and tendinous tissue lengths relative to MTU length are positively and negatively related with joint flexibility, respectively. However, these potential associations have not been experimentally demonstrated in humans in vivo.

Joint range of motion (RoM), the most common parameter for joint flexibility, is often conventionally defined as the range from the anatomical position to the angle at which subjects felt discomfort or pain [15, 16]. Unlike strain measurement in material testing, the start point of the conventionally determined joint RoM is not standardized by the joint angle at which MTU begins to resist external force (slack angle), although slack angle varies between individuals [17, 18]. Thus, it is questionable whether the potential associations between MTU architecture and joint RoM can be found when conventionally determined joint RoM (i.e., the anatomical position as the start point) is used. In other words, we can expect that the associations between MTU architecture and joint RoM would vary with how the joint RoM is determined.

It is challenging to evaluate slack angle of a muscle in vivo through joint torque measurements because the joint torque results from a composite of muscles and non-muscular tissues such as skin, ligament, and articular structures. An alternative method to evaluate individual muscle slack angle is to use ultrasound shear wave elastography (SWE) [18, 19]. This technique can noninvasively quantify a tissue mechanical property, called shear modulus, based on the propagation speed of the remotely generated shear wave within the tissue by focused ultrasound beam [20, 21]. Previous studies with this technique identified muscle slack angles from the relationship between joint angle and muscle shear modulus [17, 18, 22].

Ankle dorsiflexion RoM and/or the plantar flexors have been frequently targeted by previous studies investigating about joint flexibility, MTU extensibility, or stretching effects [e.g., 6, 7, 15–18, 23, 24]. The medial gastrocnemius (MG) is the most tensioned muscle among the plantar flexors during passive dorsiflexion when the knee is fully extended [24, 25]. Additionally, Miyamoto et al. [26] revealed that maximal dorsiflexion angle determined by the onset of pain was negatively correlated with angle-specific shear moduli of the gastrocnemii but not that of soleus. Thus, it is likely that MG is a primary contributor to dorsiflexion flexibility among the plantar flexors. The purpose of the present study was to elucidate the associations of MG-MTU architectural parameters (fascicle length, pennation angle, and tendinous tissue length) with ankle dorsiflexion RoMs determined with and without consideration of muscle slack. We hypothesized that MG-MTU architecture is associated with ankle dorsiflexion RoM determined with consideration of muscle slack, but not with the conventionally determined joint RoM, i.e., joint RoM determined without consideration of muscle slack angle.

## Materials and methods

### Subjects

Seventeen healthy males (height, 1.73 ± 0.05 m; body mass, 68.0 ± 6.3 kg; age, 22 ± 3 years; mean ± SD) participated and gave written informed consent. The subjects had no apparent neurological, orthopedic, or neuromuscular disorders. They were asked to refrain from vigorous exercise 24 hours before the experiment. The ethical approval was obtained from the Ethics Committee on Human Research of the National Institute of Fitness and Sports in Kanoya (Ethical Application Ref: H25-4-29). The present study was conducted in accordance with the Declaration of Helsinki.

### Experimental setup and protocol

The procedures of measuring ankle joint angle, fascicle length, pennation angle, muscle shear modulus of MG, and electromyography (EMG) activity were the same as those used in our previous study [23]. Subjects lay on a dynamometer (CON-TREX MJ, PHYSIOMED, Germany) bed in a prone position with their knees fully extended. The right foot was fixed to the dynamometer’s footplate firmly with non-elastic straps. The rotational axes of the ankle and the footplate were visually aligned. The ankle joint was passively dorsiflexed from 40° plantar flexion [anatomical position (i.e., the long axis of the tibia is perpendicular to the sole of the foot) defined as 0°, with positive values for dorsiflexion] to the maximal ankle joint angle at which each subject felt pain (i.e., onset of pain). The ankle joint angle was measured by using an electronic goniometer (SG110/A, Biometrics, UK) fixed to the ankle joint. In order to minimize the stretch reflex, passive ankle dorsiflexion was conducted at an angular velocity of 1°/s [16, 27]. The subjects were asked to completely relax and not to resist the footplate movement during the passive ankle dorsiflexion. Immediately before the measurement task, two cycles of the passive ankle dorsiflexion were performed to ascertain that the subjects were relaxed, and to avoid a conditioning effect of passive lengthening on the muscle and tendinous tissue stiffness [15, 23, 24]. Also, the purpose of this session was to familiarize the subjects with the RoM measurement. After the measurement task, the subjects performed a maximal voluntary isometric contraction (MVC) of plantar flexion at ankle joint angle of 0° to normalize the EMG activities during the passive ankle dorsiflexion.

### Ultrasonic measurements

**Fig 1:**
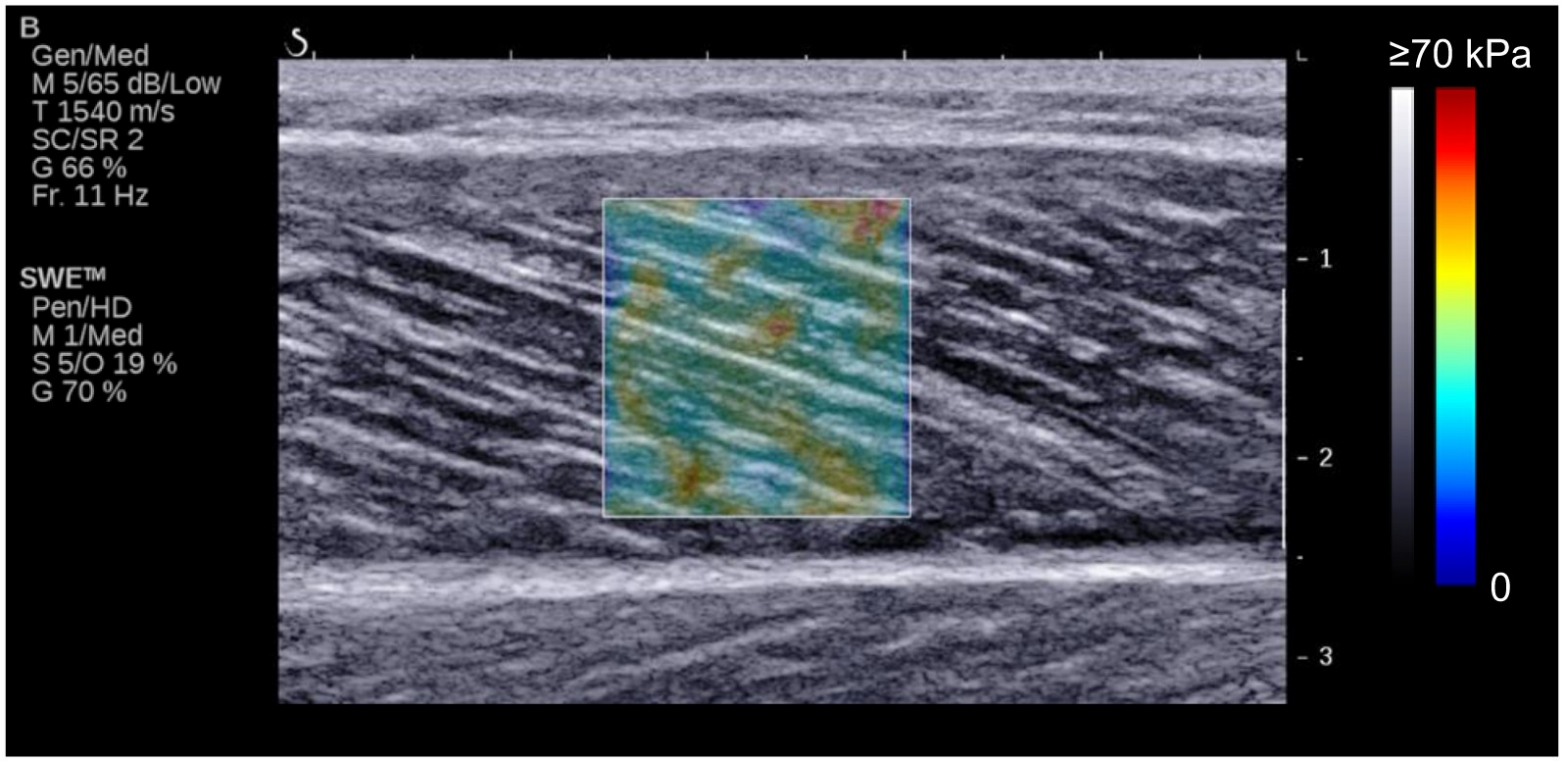
Typical example of ultrasonic image. Colored square region represents shear modulus map with the scale to the right of the figure.

To measure the fascicle length, pennation angle, and shear modulus of the MG during the passive ankle dorsiflexion task, an ultrasound SWE scanner (Aixplorer, Supersonic Imagine, France) coupled with a linear array probe (SL15-4, Supersonic Imagine, France) was used in Musculoskeletal preset. The ultrasound measurement was performed at 30% of lower leg length (from the popliteal fossa to the lateral malleolus) [23, 28]. The ultrasound probe was aligned with the fascicle plane of the MG to measure the fascicle length and pennation angle and to accurately quantify the MG shear modulus along the MG fascicle [29]. To measure the architectural parameters and shear modulus simultaneously, the opacity of ultrasound SWE image was set at 10–20%, and other settings were according to a previous study [23]: SWE optimization = Pen, image optimization = HD, persistence = Med, spatial smoothing = 5 (Fig 1). The B-mode and SWE images were acquired at 11Hz and 1 Hz, respectively.

### EMG measurement

EMG activities were measured to assure muscle relaxation during the passive ankle dorsiflexion task. Pre-amplified bipolar active surface EMG electrodes (electrode shape: parallel-bar, size: 1 mm width × 10 mm length, inter-electrode distance: 10 mm; DE-2.1, DELSYS, USA) with band-pass filtering between 20 and 450 Hz (Bagnoli 8 EMG System, DELSYS, USA) were placed over the bellies of the MG, the lateral gastrocnemius and soleus along the fascicle direction. Prior to this, shaving with razors, abrasion with sandpaper, and cleaning with alcohol were performed. The reference electrode was placed on the left medial malleolus.

### Data collection and analysis

In the present study, all reported ankle joint angles refer to the angle assessed with the goniometer. The ankle joint angle and EMG data were stored on a personal computer via a 16-bit analog-to-digital converter (PowerLab 16/35, ADInstruments, Australia) with a sampling frequency of 1 kHz. The data were synchronized with B-mode and SWE recordings by matching the clock time of the personal computer and SWE scanner.

**Fig 2:**
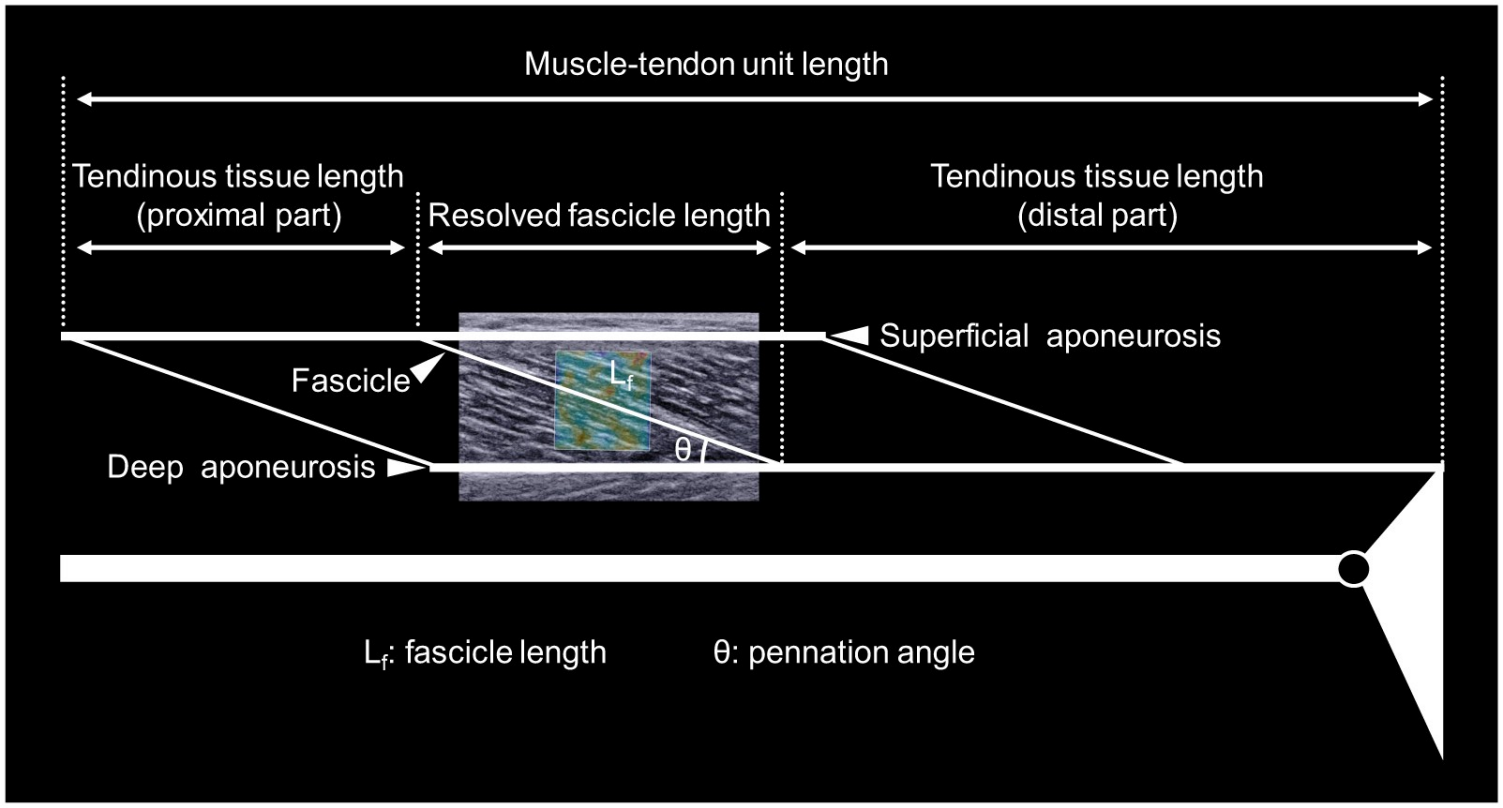
Schematic representation of definitions of the muscle-tendon unit architectural parameters.

The color map opacities of ultrasonic videos were reset at 0% and 100% for the subsequent analyses of muscle and tendinous tissue architecture and shear modulus, respectively. Then, videos were exported as “mp4” format and sequenced in “png” format. The B-mode image data analyses were performed by standard image processing software (ImageJ, NIH, USA). Fascicle length was assessed as the length of the straight line between the intersection points of the fascicle and superficial and deep aponeuroses (Fig 2). If fascicle was not visible entirely in the image, linear extrapolation was conducted to estimate the fascicle length [23, 30]. The angle between fascicle and deep aponeurosis was defined as pennation angle. The resolved fascicle length was calculated as the fascicle length multiplied by the cosine of the pennation angle [16, 31]. The length of MG-MTU was estimated from the ankle and knee joints angles and lower leg length according to the anthropometric model provided by Grieve et al. [32]. The tendinous tissue length was estimated by subtracting the resolved fascicle length from the MTU length [6, 33]. The ratios of the resolved fascicle length and tendinous tissue length to MTU length were calculated as the relative muscle and tendinous tissue lengths to MG-MTU length, respectively. The architectural parameters of MG-MTU were analyzed at the slack angle of MG (see below) and at the anatomical position.

**Fig 3:**
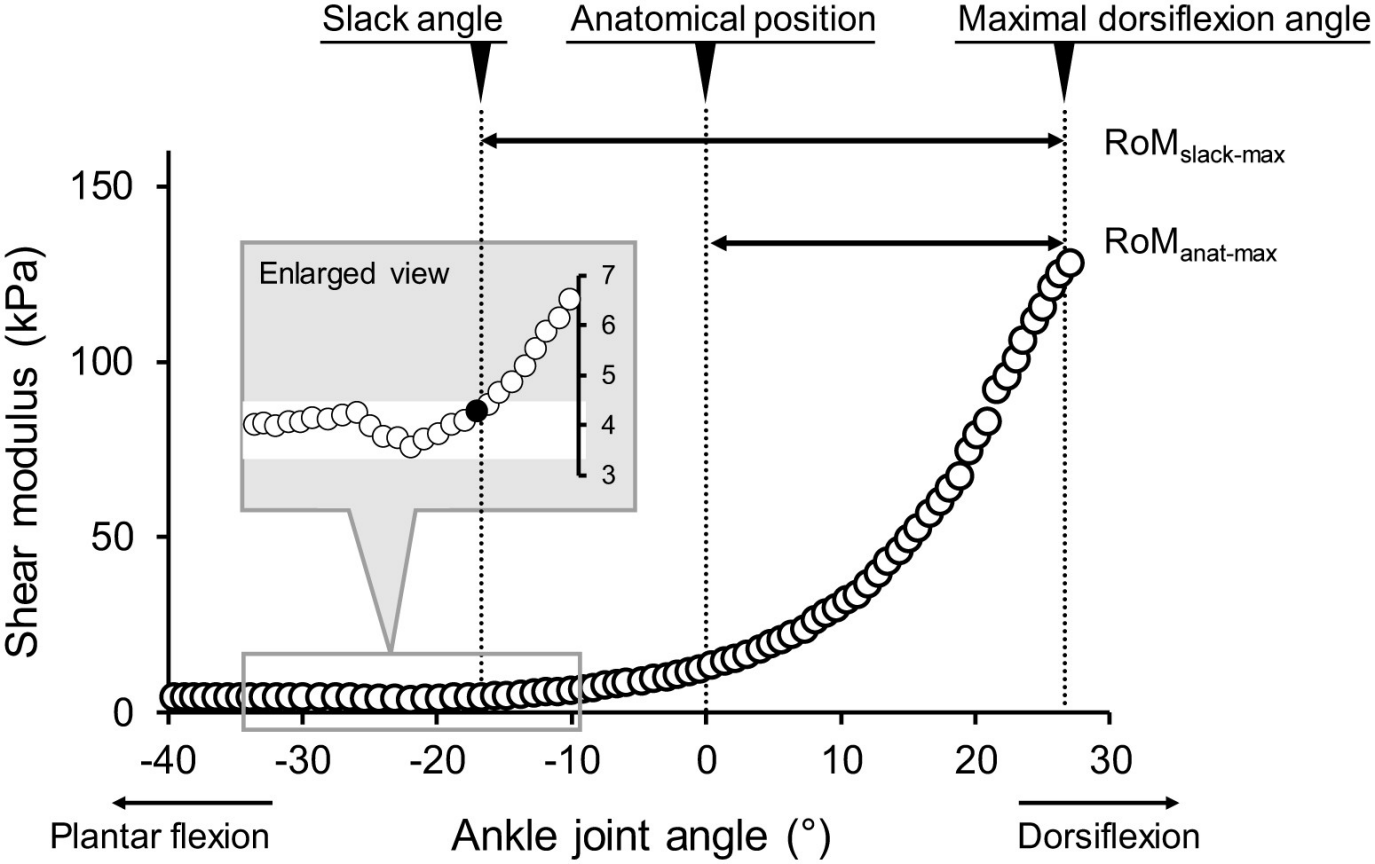
Typical example of the ankle joint angle-shear modulus relationship. Schematics defining two ranges of motion (RoM_anat-max_ and RoM_slack-max_) are shown. Enlarged view represents how to detect slack angle. anat: anatomical position, max: maximal joint angle, RoM: range of motion, slack: slack angle.

For SWE images, data analyses were performed by the image processing software (ImageJ, NIH, USA). Care was taken to select a region of interest (ROI) of shear modulus map as large as possible with exclusion of aponeuroses and subcutaneous adipose tissues for each image. An average value of the shear modulus over the ROI was calculated for each image. According to previous studies [22, 23], the slack angle of MG was determined from the ankle joint angle-shear modulus relationship as the first increase above the variation in shear modulus (Fig 3). All measurements and analyses of B-mode and SWE data were performed by an examiner with more than 3 years of experience. In the present study, ankle dorsiflexion flexibility was evaluated as the following two RoM: 1) RoM between the anatomical position and the maximal dorsiflexion angle (RoM_anat-max_), 2) RoM between the slack angle and the maximal dorsiflexion angle (RoM_slack-max_) (Fig 3). For EMG data, the root-mean-square values (EMG-RMSs) were calculated during passive ankle dorsiflexion for each muscle of the triceps surae. Then, the EMG-RMS values of each muscle were normalized to those for 1 s during MVC.

### Statistics

A priori power analysis was performed to calculate the minimum sample size based on an assumed type 1 error of 0.05 and a statistical power of 0.8 (type 2 error rate of 0.2). Effect size was assumed to be 0.65, based on previous studies [26, 34], which investigated the correlations of joint RoM with muscle architecture. The analysis revealed that the minimum sample size was 13. Descriptive data are expressed as means and SDs. Statistical software (SPSS Statistics 21, IBM Japan, Japan) was used for statistical analyses. Pearson’s product-moment correlation coefficients were calculated to examine the relations between MTU architectural parameters (fascicle length, resolved fascicle length, pennation angle, tendinous tissue length, muscle length relative to MTU length, and tendinous tissue length relative to MTU length) measured at the slack angle or anatomical position, and ankle dorsiflexion RoM (RoM_anat-max_, RoM_slack-max_). P < 0.05 was considered statistically significant.

## Results

RoM_anat-max_ was 25.8 ± 6.3°, and RoM_slack-max_ was 44.0 ± 8.1°. There was a significant (but moderate) correlation between RoM_anat-max_ and RoM_slack-max_ (r = 0.722, P = 0.001). Table 1 shows descriptive data on the MG-MTU architectural parameters at the MG slack angle and anatomical position.

**Table 1:** Descriptive data of muscle-tendon unit architectural parameters at slack angle and anatomical position.

	Slack angle	Anatomical position
Fascicle length (mm)	52.7 ± 6.2	66.9 ± 10.8
Pennation angle (°)	24.2 ± 2.7	18.6 ± 3.5
Resolved fascicle length (mm)	48.1 ± 5.9	63.4 ± 11.0
Tendinous tissue length (mm)	359.7 ± 31.7	359.0 ± 33.2
MTU length (mm)	407.8 ± 28.4	422.4 ± 27.4
Muscle length relative to MTU length	0.12 ± 0.02	0.15 ± 0.03
Tendinous tissue length relative to MTU length	0.88 ± 0.02	0.85 ± 0.03

MTU: muscle-tendon unit.

The MG-MTU architectural parameters measured at the anatomical position showed no significant correlations with RoM_anat-max_ or RoM_slack-max_ (Table 2). Regarding the MG-MTU architectural parameters measured at the slack angle, the resolved fascicle length, muscle length relative to MTU length, and tendinous tissue length relative to MTU length significantly correlated with RoM_slack-max_ (Table 3). In contrast, none of the MG-MTU architectural parameters measured at the slack angle showed a significant correlation with RoM_anat-max_.

**Table 2:** Correlation coefficients between the muscle-tendon unit architectural parameters at anatomical position and ankle dorsiflexion ranges of motion.

Architectural parameters	RoM_anat-max_	RoM_slack-max_
Fascicle length	-0.123	0.449
	(P = 0.639)	(P = 0.071)
Pennation angle	0.142	-0.253
	(P = 0.586)	(P = 0.328)
Resolved fascicle length	-0.129	0.450
	(P = 0.622)	(P = 0.070)
Tendinous tissue length	-0.087	-0.416
	(P = 0.739)	(P = 0.096)
MTU length	-0.157	-0.325
	(P = 0.547)	(P = 0.203)
Muscle length relative to MTU length	-0.104	0.422
	(P = 0.690)	(P = 0.091)
Tendinous tissue length relative to MTU length	0.104	-0.422
	(P = 0.690)	(P = 0.091)

MTU: muscle-tendon unit, RoM_anat-max_: range of motion between the anatomical position and the maximal dorsiflexion angle, RoM_slack-max_: range of motion between the slack angle and the maximal dorsiflexion angle.

**Table 3:** Correlation coefficients between the muscle-tendon unit architectural parameters at slack angle and ankle dorsiflexion ranges of motion.

Architectural parameters	RoM_anat-max_	RoM_slack-max_
Fascicle length	0.002	0.478
	(P = 0.994)	(P = 0.052)
Pennation angle	0.068	-0.173
	(P = 0.795)	(P = 0.507)
Resolved fascicle length	-0.011	0.491*
	(P = 0.967)	(P = 0.045)
Tendinous tissue length	-0.121	-0.461
	(P = 0.643)	(P = 0.062)
MTU length	-0.137	-0.414
	(P = 0.599)	(P = 0.099)
Muscle length relative to MTU length	0.033	0.506*
	(P = 0.900)	(P = 0.038)
Tendinous tissue length relative to MTU length	-0.033	-0.506*
	(P = 0.900)	(P = 0.038)

* Statistically significant at P < 0.05.

MTU: muscle-tendon unit, RoM_anat-max_: range of motion between the anatomical position and the maximal dorsiflexion angle, RoM_slack-max_: range of motion between the slack angle and the maximal dorsiflexion angle.

EMG-RMSs of MG, the lateral gastrocnemius and the soleus during passive ankle dorsiflexion were 1.2 ± 0.8, 0.9 ± 0.9, and 1.8 ± 1.6%MVC, respectively, indicating that the muscles were relaxed during the task.

## Discussion

We aimed to clarify the associations of MG-MTU architecture with dorsiflexion RoM determined with and without consideration of muscle slack. The resolved fascicle length, muscle length relative to MTU length, and tendinous tissue length relative to MTU length measured at the slack angle were significantly correlated with the joint RoM determined with consideration of slack angle (i.e., RoM between the slack angle and the maximal dorsiflexion angle; RoM_slack-max_). In contrast, no significant correlations were observed MG-MTU architecture measured at the slack angle and conventionally-determined joint RoM (i.e., RoM_anat-max_) and between MG-MTU architecture measured at the anatomical position and RoM_anat-max_ (conventionally-determined joint RoM without consideration of slack angle) or RoM_slack-max_ (RoM with consideration of slack angle). These results support our hypothesis that MTU architecture would be associated with joint RoM determined with, but not without, consideration of muscle slack, and suggest that long fascicle or short tendinous tissue has beneficial effects on joint flexibility.

It is theoretically [11, 12] and experimentally [6, 13] implied that long fascicle is advantageous to joint flexibility through its extensibility. Nevertheless, we failed to find a significant correlation of fascicle length with RoM_anat-max_. This is probably because RoM_anat-max_ was determined without consideration of slack angle. In the anatomical position of the ankle joint with the knee extended, MG-MTU was already tensioned and beyond the slack, as observed in the present and previous researches [17, 18, 25]. Considering the individual difference in slack angle, the amount of MG-MTU stretch from its slack length to the anatomical position should vary among the subjects. Thus, MTU architecture and RoM determined using parameters measured at the anatomical position cannot precisely reflect the individual difference in MTU extensibility. In other words, it may not make sense to associate between MTU architecture and joint flexibility unless these are considered with slack angle. Indeed, the present study demonstrated a significant correlation of resolved fascicle length with RoM when the individual difference in slack angle was taken into account (i.e., ROM_slack-max_).

Several studies have suggested that long tendinous tissues would be more compliant than short tendinous tissues [35–37]. Thus, it is theoretically possible that tendinous tissue length would positively correlate with joint RoM. However, in practice, there was no significant correlation between tendinous tissue length and RoM with or without consideration of slack angle. This could be explained by the fact that MTU length is limited in vivo and accordingly it is impossible that long fascicle and tendinous tissue are simultaneously present. Additionally, muscle is much more compliant than tendinous tissue in resting condition [14]. Considering the present findings that MTU length was not correlated with ROM_ana-max_ or ROM_slack-max_ and that muscle and tendinous tissue lengths relative to MTU length were positively and negatively correlated with RoM_slack-max_, respectively, it is beneficial to arrange serially long fascicle and short tendinous tissue in the limited MTU length for yielding greater joint flexibility.

MTUs with larger pennation angle are considered to be easily elongated, based on the concept of gear effect [38, 39]. However, pennation angle was not correlated with ROM_ana-max_ or ROM_slack-max_. One of the reasons might be due to a small contribution of pennation angle to joint flexibility. Assuming that fascicle length is equivalent for all subjects, the coefficient of variation of pennation angle of the present study contributes by only ~1.5% to that of the change in MTU length (calculated from the change in cosine of fascicle length) in the case of the same fascicle strain. Conversely, assuming that pennation angle is equivalent for all subjects, the coefficient of variation of fascicle length has a contribution by ~8% to that of the change in MTU length under the same fascicle strain. Although these are just calculated in simple models, it is suggested that the impact of pennation angle on MTU extensibility is weaker ~6 times than that of fascicle length. Similarly, Herbert et al. [40] reported that, for MG, the contribution of pennation angle to MTU elongation was much smaller than that of fascicle length or tendinous tissue length. Taken together, the impact of pennation angle of MG on the ankle dorsiflexion RoM would be negligibly small although pennation angle theoretically influences MTU extensibility through the gear effect.

Although only males were recruited in the present study, dorsiflexion RoM [26], mechanical properties [26], and architectural parameters of MG [41] differ by sex. Additionally, Miyamoto et al. [26] suggested that maximal dorsiflexion angle of females is dependent on stretch tolerance rather than mechanical properties of the triceps surae, unlike that of males. Hence, it is unclear that the present findings hold true for females. On the other hand, previous studies reported that female athletes with high joint flexibility (rhythmic gymnasts and ballet dancers) had longer MG fascicle than female volleyball athletes [42] or non-stretching controls [43]. These reports are in line with our results. Therefore, even in females, long fascicle can be beneficial for joint flexibility, while contribution of MTU architecture to joint flexibility might differ by sex.

There are limitations of the present study. First, we only measured the shear modulus at the single site of MG. However, a previous study indicated that there was no intramuscular differences in slack angle [25]. Secondly, we measured the tendinous tissue as a complex of aponeurosis and free tendon. Because mechanical properties of aponeurosis differ from those of free tendon [44], the influence of architecture on RoM can also vary between them. Thus, future study is warranted to assess the architecture of aponeurosis and tendon separately.

## Conclusions

The present study demonstrated utilizing ultrasound SWE that MTU architecture (resolved fascicle length and relative muscle/tendinous-tissue length to MTU length) is potentially associated with joint RoM. We propose that MTUs with long fascicle and short tendinous tissue is advantageous with respect to joint flexibility. Nevertheless, such advantages of muscle-tendon architecture cannot be found unless joint RoM is assessed with consideration of muscle slack.

## Supporting information

S1 Table Data set used for statistical analyses in this paper. (XLSX)
